# Retinal Degeneration In A Mouse Model Of CLN5 Disease Is Associated With Compromised Autophagy

**DOI:** 10.1038/s41598-017-01716-1

**Published:** 2017-05-09

**Authors:** Henri Leinonen, Velta Keksa-Goldsteine, Symantas Ragauskas, Philip Kohlmann, Yajuvinder Singh, Ekaterina Savchenko, Jooseppi Puranen, Tarja Malm, Giedrius Kalesnykas, Jari Koistinaho, Heikki Tanila, Katja M. Kanninen

**Affiliations:** 1 A.I. Virtanen Institute for Molecular Sciences, University of Eastern Finland, Kuopio, Finland; 2 Experimentica Ltd., Kuopio, Finland; 3 Research and Development Centre for Ophthalmic Innovations (SILK), Department of Ophthalmology, University of Tampere, Tampere, Finland; 4 Department of Pharmacology, School of Medicine, Case Western Reserve University, 10900 Euclid Avenue, Cleveland, OH 44106 USA

## Abstract

The Finnish variant of late infantile neuronal ceroid lipofuscinosis (CLN5 disease) belongs to a family of neuronal ceroid lipofuscinosis (NCLs) diseases. Vision loss is among the first clinical signs in childhood forms of NCLs. Mutations in *CLN5* underlie CLN5 disease. The aim of this study was to characterize how the lack of normal functionality of the CLN5 protein affects the mouse retina. Scotopic electroretinography (ERG) showed a diminished c-wave amplitude in the CLN5 deficient mice already at 1 month of age, indicative of pathological events in the retinal pigmented epithelium. A- and b-waves showed progressive impairment later from 2 and 3 months of age onwards, respectively. Structural and immunohistochemical (IHC) analyses showed preferential damage of photoreceptors, accumulation of autofluorescent storage material, apoptosis of photoreceptors, and strong inflammation in the CLN5 deficient mice retinas. Increased levels of autophagy-associated proteins Beclin-1 and P62, and increased LC3b-II/LC3b-I ratio, were detected by Western blotting from whole retinal extracts. Photopic ERG, visual evoked potentials, IHC and cell counting indicated relatively long surviving cone photoreceptors compared to rods. In conclusion, CLN5 deficient mice develop early vision loss that reflects the condition reported in clinical childhood forms of NCLs. The vision loss in CLN5 deficient mice is primarily caused by photoreceptor degeneration.

## Introduction

The neuronal ceroid lipofuscinoses (NCLs), also known as Batten disease, are the most common neurodegenerative disease group among children. The overall incidence of NCLs in the USA is estimated to be 1:12 500, and certain forms of NCL are relatively frequent in Northern Europe^1^. As the NCLs are difficult to diagnose the incidence may be even higher. This group of disorders is associated with mutations in at least 13 different genes^2^, causative of several distinct NCL disease subtypes called the CLNs (CLN1-CLN8 and CLN10-CLN14). The cellular localization of all protein products of NCL associated genes is not well established (e.g. *CLN3*), but principally represent soluble lysosomal enzymes, polytopic membrane proteins localized in lysosomes or in the endoplasmic reticulum (ER), or synaptic vesicle associated proteins^3^. Although the protein products have differential functions and intracellular locations, all subtypes of NCL generally (*i.e*. childhood forms), but not entirely (*i.e*. adulthood forms), lead to similar clinical symptoms: vision loss, motor and mental deterioration, spontaneous seizures, and premature death^1^. Vision loss is often among the first detectable clinical signs in childhood forms of NCLs and ophthalmological examinations can be used to aid in the clinical diagnosis of the disease^4^. A characteristic pathological feature of the NCLs is the accumulation of autofluorescent storage material, lipofuscin-like ceroids, into lysosomes^5^. According to current opinion, accumulation of lysosomal storage material may not be the primary cause of NCL pathology and the exact disease mechanisms remain unresolved^6^. To date, there is no drug regulatory agency (*e.g*. FDA, EMA) approved disease modifying pharmacotherapies available for NCLs^7^. However, several phase I-II clinical trials using gene, stem cell or enzyme replacement therapies are ongoing (see *e.g*. NCT00151216, NCT01414985, NCT02725580, NCT02678689, NCT01586455; ClinicalTrials.gov).

Several mouse models have been developed that recapitulate different forms of human NCLs^8^. With regard to the retina, the majority of the mouse models tested so far show pathology primarily at the outer retina where the photoreceptors reside^9–13^. CLN6 deficient mice display the most robust retinal phenotype within the assessed NCL mouse models to date. In these mice, the retinopathy begins briefly after eye opening and is characterized by early onset photoreceptor death and retinal inflammation^9, 12^. In another NCL mouse model, Wavre-Shapton *et al*. (2015) recently showed defects in phagosome processing and autophagy in the retinal pigmented epithelium (RPE)^14^. Several research groups have earlier shown compromised autophagy in other organs of NCL mouse models^15–18^. In addition, NCLs naturally affect some breeds of sheep and dogs which have been harnessed as large animal models of NCLs^19–24^. With respect to vision, Dalmatian dogs, English Setters, Tibetan Terriers and Border Collies affected with NCL do not well resemble childhood form of NCLs as the progression of retinopathy in these models is slow or even absent^20–22^. Instead, New Hampshire Sheep, Polish Owczarek Nizinny (PON) dogs and Dachshunds dogs affected with NCL do manifest rather strong retinopathy^20, 23, 24^. As in mice, also in ovine and canine models of NCLs the photoreceptors seem to be primarily affected^20, 21, 24–26^. Accordingly, the retinal degeneration in most forms of human NCLs is believed to start at the outer segments of photoreceptors^5^.

Careful characterization of retinopathy in different NCL subtypes is warranted since it may eventually answer the question of whether retinopathy in several distinct forms of NCLs have a common mechanism, and whether this mechanism shares a link to brain pathology in NCLs. Indeed, the superior accessibility of the retina for experimental modulation and imaging makes the retina an attractive window to the brain^27^. Progressive vision loss has been described previously in CLN5 deficient mice based on a simple behavioral test^28^. In this study, we characterized the retinal phenotype of CLN5 deficient mice thoroughly for the first time.

## Results

### Early rod-mediated functional deficits in CLN5 deficient mouse retinas

**Figure 1:**
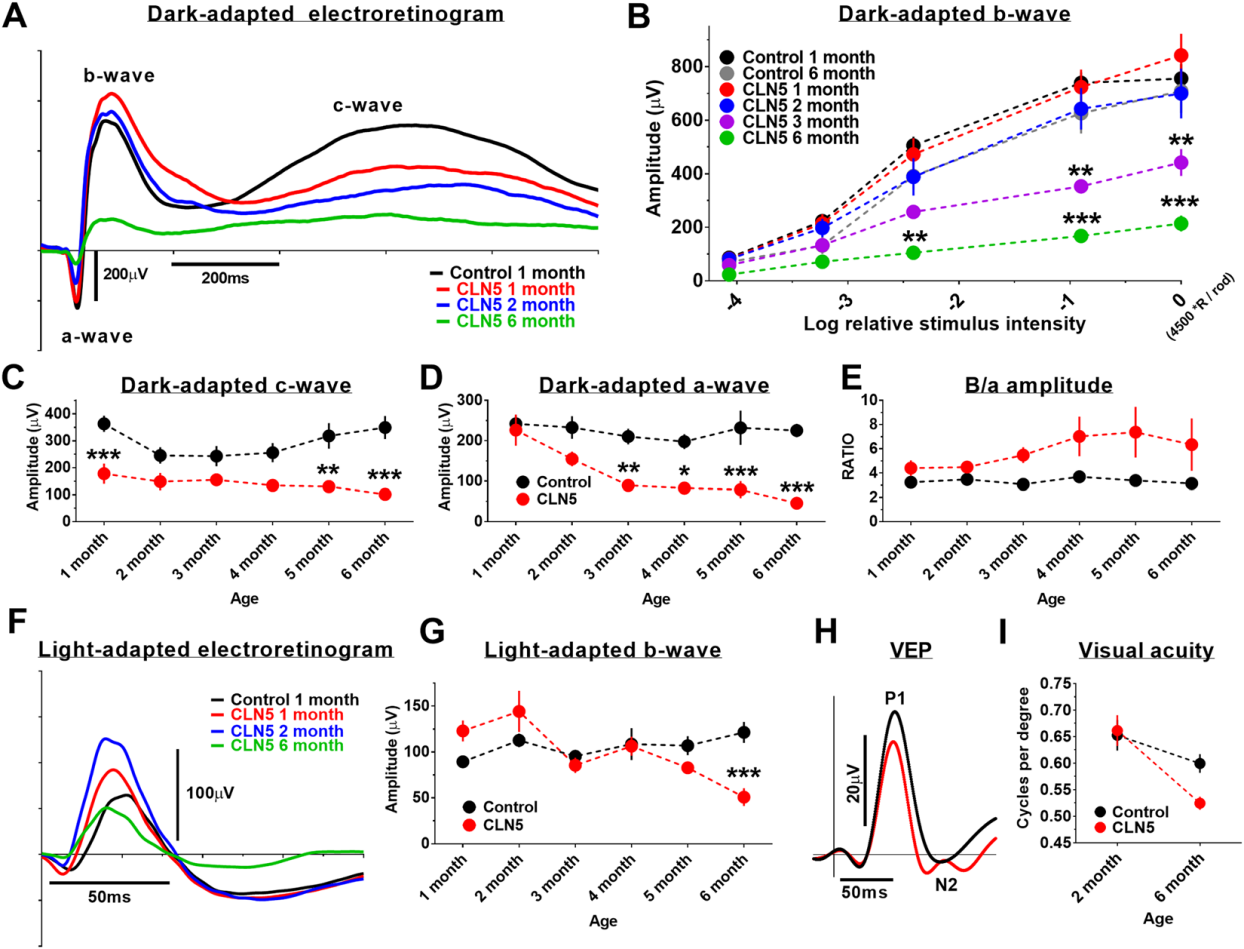
Progressive decline in retinal function and cortical visual acuity in CLN5 deficient mice. (**A**) group averaged band- filtered (70–150 Hz) scotopic ERG waveforms (at log 0) in 1-month-old control and CLN5 deficient mice (groups sizes: control 1 month, n = 12; control 2 month, n = 10, control 3–6 month, n = 6; CLN5 1 month, n = 9; CLN5 2–6 month, n = 6–8). (**B**) scotopic b-wave amplitudes over age. Note that all age points are not shown for clarity of presentation. The maximum stimulus intensity (log 0) yielded ~4500 photoisomerizations per rod. (**C,D**) scotopic c- and a-wave amplitudes (at log 0) in control and CLN5 deficient mice over age, respectively. (**E**) scotopic b-wave/a-wave amplitude ratio at log 0 flash. (**F**) group averaged band- filtered (70–150 Hz) photopic ERG waveforms (at log 0) in 1-month-old control and 1-month, 2-month and 6-month-old CLN5 deficient mice. (**G**) photopic b-wave amplitudes (at log 0) in control and CLN5 deficient mice over age. (**H**) Representative group averaged VEP waveforms in response to middle size (0.192 CPD) pattern stimulus in 6-month-old mice. (**I**) Cortical visual acuity as determined by pattern VEPs (n = 11 per genotype). Bonferroni multiple comparison test: *p < 0.05, **p < 0.01, ***p < 0.001.

Scotopic electroretinogram (ERG) a- and b-wave amplitudes, depicting rod-function, were normal at 1 month of age in CLN5 deficient mice. Instead, the c-wave amplitude was already smaller in CLN5 deficient than in control mice (F_1,72_ = 64.61, p < 0.001, Fig. 1A,C). The a-wave appeared reduced at 2 months of age reaching statistical significance at 3 months of age (F_1,79_ = 56.7, p < 0.001, Fig. 1D). The decrease in scotopic b-wave amplitude started at 3 months of age (F_6,235_ = 34.9, p < 0.001; Fig. 1B). B-wave/a-wave amplitude ratio tended to be higher in CLN5 deficient mice over the course of the study (F1,73 = 16.2, p < 0.001; Fig. 1E). Retinal function under photopic conditions, depicting cone-function, remained similar in control and CLN5 deficient mice for several months, but eventually also the photopic ERG b-wave amplitude started to decline in CLN5 deficient mice at the age of 5 months (Fig. 1F,G), although the difference between groups reached statistical significance only at 6 months of age (interaction: F_5,79_ = 6.3, p < 0.001). Correspondingly, medium-to-long (M/L) wave length cone density and macroscopic appearance of M/L cone outer segments (COS) seemed normal in CLN5 deficient mice at 3 months of age, whereas at 6 months the cone density started to show a trend towards decline and COS appeared shrunken (F_1,15_ = 4.1, p = 0.06; Supplementary Fig. 1). Visual acuity as determined by the VEP technique was similar between the genotypes at 2 months of age, but showed a trend towards decline in CLN5 deficient mice at 6 months of age (interaction: F_1,20_ = 3.00, p = 0.10, Fig. 1I). The VEP implicit time remained normal in CLN5 deficient mice upon aging (quantitative data not shown, but see representative averaged VEP waveform in Fig. 1H), indicating uninjured optic nerves.

### Outer nuclear layer thickness follows the time sequence of rod dysfunction

**Figure 2:**
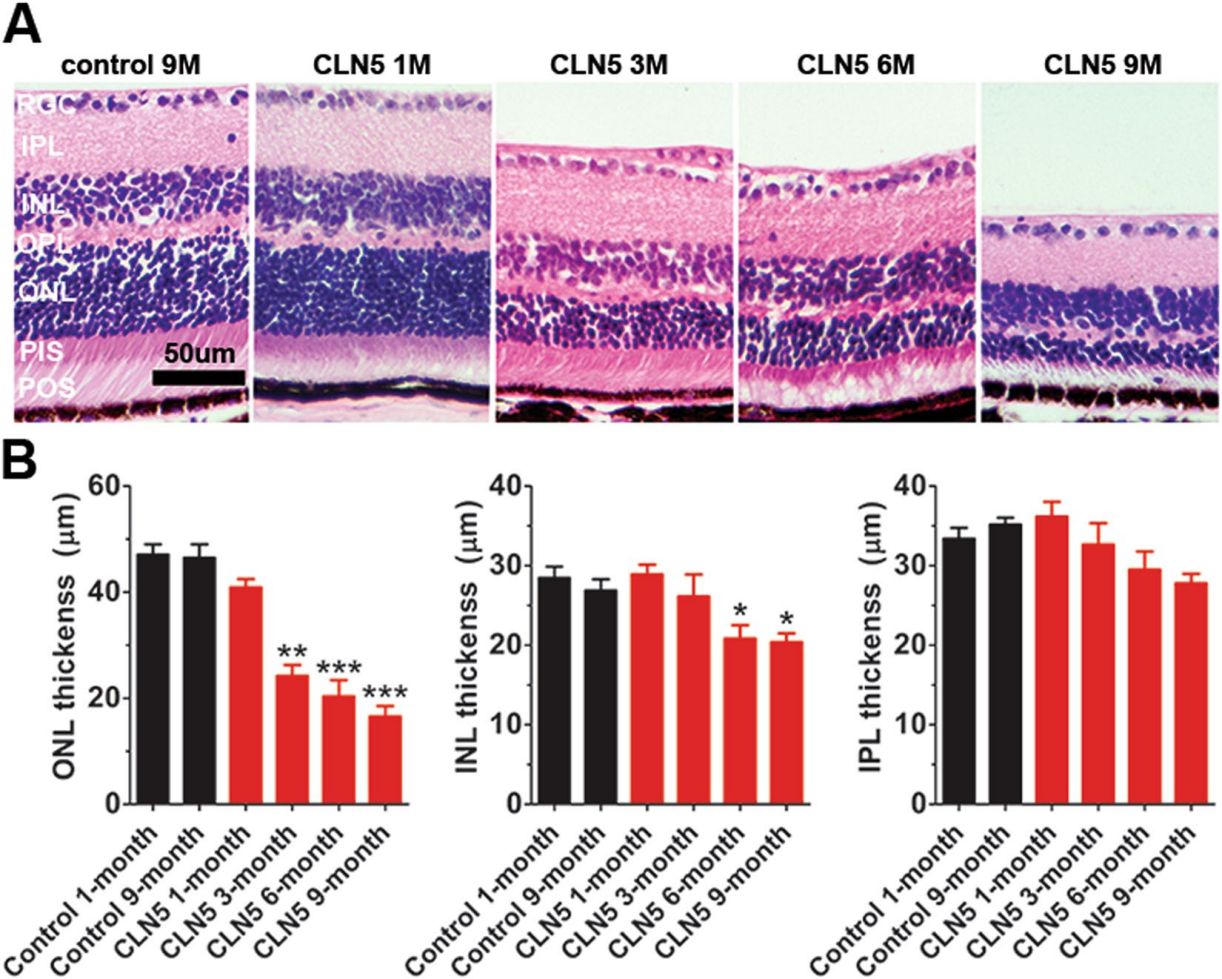
Robust photoreceptor death in CLN5 deficient mice. (**A**) Representative H&E stained retinal sections. At 1 month of age CLN5 deficient retina represents completely normal morphology. Also at 3 month of age the CLN5 deficient retina has normal appearance but the ONL is drastically thinned. The pathology progressed so that the POS started to become disorganized and disappeared, and at the late state of disease at 9 months of age even PIS died out. The inner retinal compartments remained normal appearance over the course of the follow up. (**B**) Statistical analysis of ONL, INL and IPL thicknesses (control 1 month, n = 4–5; control 9 month, n = 4; CLN5 1 month, n = 4; CLN5 3 month, n = 5; CLN5 6 month, n = 6–7; CLN5 9 month, n = 6) showed that ONL was primarily affected in CLN5 deficient mice retinas with early onset whereas INL and IPL showed slowly progressive thinning. Tukey’s posthoc test: *p < 0.05, **p < 0.01, ***p < 0.001. RGC, retinal ganglion cells; IPL, inner plexiform layer; INL, inner nuclear layer; OPL, outer plexiform layer; ONL, outer nuclear layer; PIS, photoreceptor inner segments; POS, photoreceptor outer segments.

Anatomical inspection of retinal sections confirmed that the outer nuclear layer (ONL), where photoreceptors reside, was primarily affected in CLN5 deficient mice (Fig. 2A). The ONL thickness appeared reduced in CLN5 deficient mice already at the age of 1 month (mean difference between CLN5 deficient and control samples was 6.19 µm). At the age of 3 months, the ONL was drastically thinned in CLN5 deficient compared to the control mice (F_5,25_ = 31.75, p < 0.001; Fig. 2A,B). The morphology of photoreceptor outer segments (POS) in CLN5 deficient mice looked relatively normal until 3 months of age, but was clearly damaged at 6 months and even further at 9 months of age. In addition, photoreceptor inner segments were clearly damaged in 9-month-old CLN5 deficient mice. In contrast, the inner retinal compartments, *i.e*. interneuron and ganglion cell layers, showed a normal appearance at all age points studied (Fig. 2A). However, inner nuclear layer (INL) also became thinner in CLN5 deficient mice eventually at the age of 6 months (F_5,24_ = 4.96, p < 0.05; Fig. 2B). Likewise, the inner plexiform layer (IPL) showed a slow progressive decrease in CLN5 deficient mice (F_5,23_ = 2.88, p < 0.05).

### Accumulation of autofluorescent storage matrial, altered autophagy, apoptotic photoreceptor cells, and strong inflammation in CLN5 deficient mouse retinas

**Figure 3:**
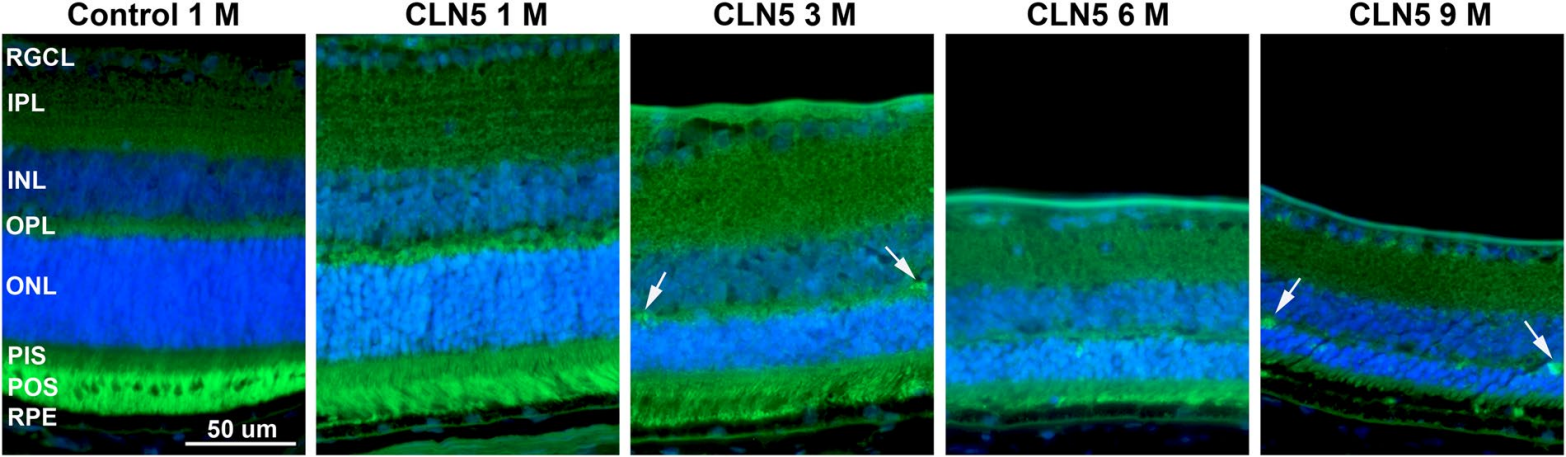
Increased autofluorescence (AF) in CLN5 deficient mouse retinas. Images were captured with a fluorescent microscope using auto-exposure. CLN5 deficient retinas seem to display stronger diffuse AF throughout the retina as compared to wild-type control. AF aggregates started to appear from 3 month of age onwards particularly into the OPL (arrows), which is more obvious in Supplementary Fig. 2. RGCL, retinal ganglion cell layer; IPL, inner plexiform layer; INL, inner nuclear layer; OPL, outer plexiform layer; ONL, outer nuclear layer; PIS, photoreceptor inner segments; POS, photoreceptor outer segments; RPE, retinal pigmented epithelium.

NCLs are characterized by accumulation of autofluorescent storage material. We inspected retinal cross-sections and whole mounts by fluorescence microscope and observed increase in autofluorescence (AF) in CLN5 deficient retinas compared to controls (Fig. 3 and Supplementary Fig. 2). Diffuse AF seemed increased throughout the CLN5 deficient retinas (Fig. 3). AF aggregates accumulated as aggregates particularly into the outer plexiform layer (Supplementary Fig. 2).

**Figure 4:**
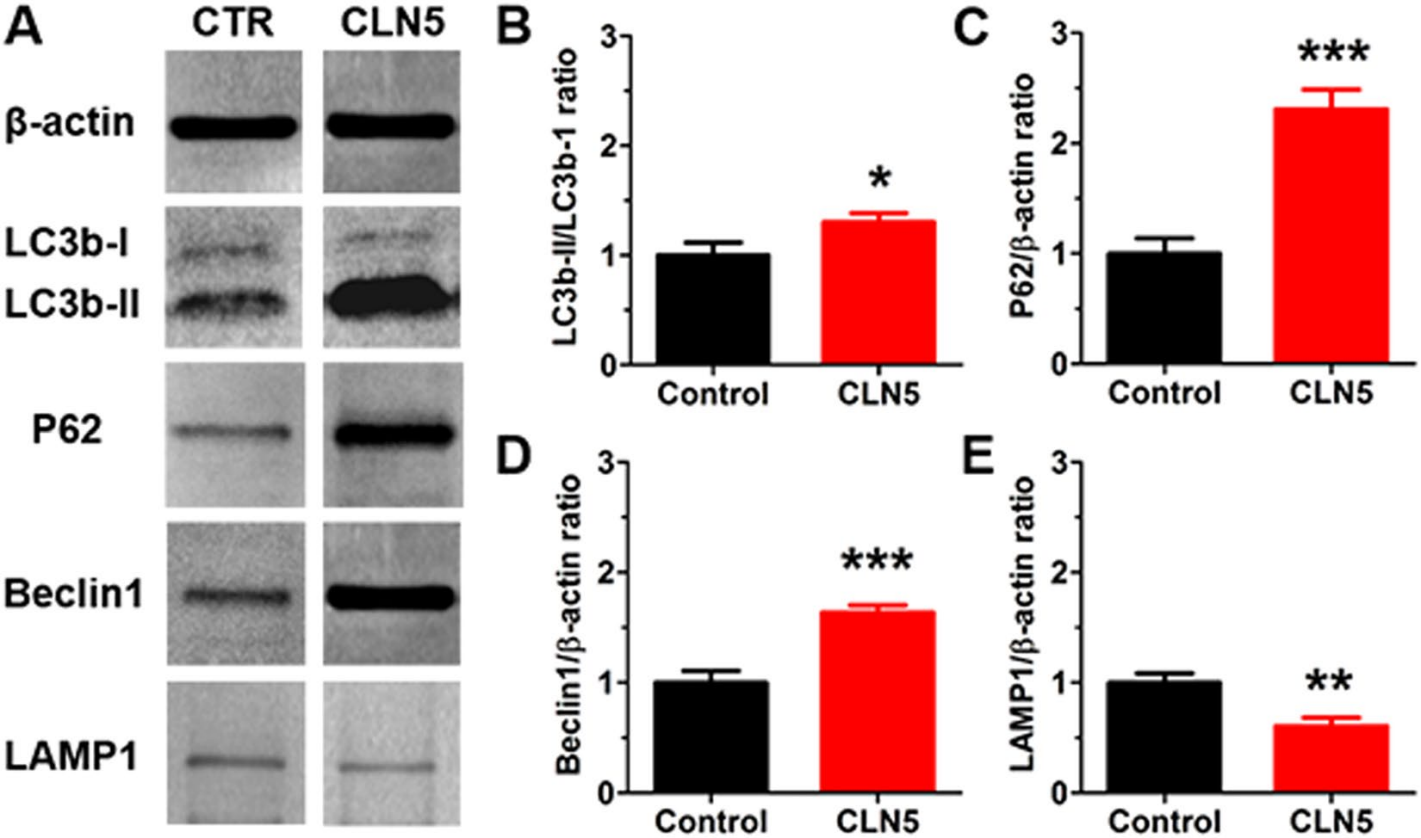
Altered autophagy in 6-month-old CLN5 deficient mouse retinas. (**A**) Representative immunoblots. (**B**) LC3-I to LC-II conversion was increased 1.3-fold in CLN5 deficient retinas (n = 10 per genotype). (**C,D**) P62 level was increased 2.3-fold, and Beclin-1 level 1.6-fold in CLN5 deficient retinas (n = 7 and 8 per genotype, respectively). (**E**) LAMP1 level was decreased in CLN5 deficient retinas and was 0.6-fold lower when compared to control levels (n = 8 per genotype). The protein level ratios in CLN5 deficient samples were compared to normalized control values in all analyses (**B–E**). *p < 0.05, **p < 0.01, p < 0.001; t-test.

By Western blotting we detected markedly increased levels of autophagy-associated proteins Beclin1 and P62 in 6-month-old CLN5 deficient mouse retinas relative to sex and age-matched control retinas (Beclin-1, 1.6-fold increase, t_14_ = 4.98, p < 0.001; P62, 2.3-fold increase, t_14_ = 5.71, p < 0.001; Fig. 4). Conversion of LC3b-I to LC3b-II was also increased in CLN5 deficient retinal samples (LC3b-II/LC3b-I ratio, 1.3-fold increase, t_18_ = 2.11, p < 0.05). The level of the lysosomal membrane protein LAMP1 was reduced in CLN5 deficient retinal samples when compared to controls (LAMP1, 0.6-fold reduction, t_14_ = 3.47, p < 0.01).

**Figure 5:**
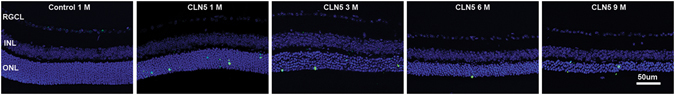
Apoptotic photoreceptor nuclei in CLN5 deficient mice retinas. Representative fluorescent z-stack images of TUNEL staining (green) with DAPI counter stain (blue, cell nuclei). At 1 month of age apoptotic cells were frequent in CLN5 deficient mice samples but the amount decreased with diminishing amount of surviving photoreceptors (see Supplementary Fig. 3). Apoptotic cells were restricted to the ONL. RGCL, retinal gangion cell layer; INL, inner nuclear layer; ONL, outer nuclear layer.

Apoptotic cells were abundant in the ONL in CLN5 deficient mice already at 1-month of age (Fig. 5). The relative count of TUNEL positive cells appeared similar in all ages investigated (1–9 months) when the progressive loss of photoreceptors was taken into account (Supplementary Fig. 3). The TUNEL positive cells were virtually restricted to the ONL. No positive TUNEL cells were seen in the ONL of control mouse samples at any age; however, there were some sporadic TUNEL cells in the RGCL (data not shown).

**Figure 6:**
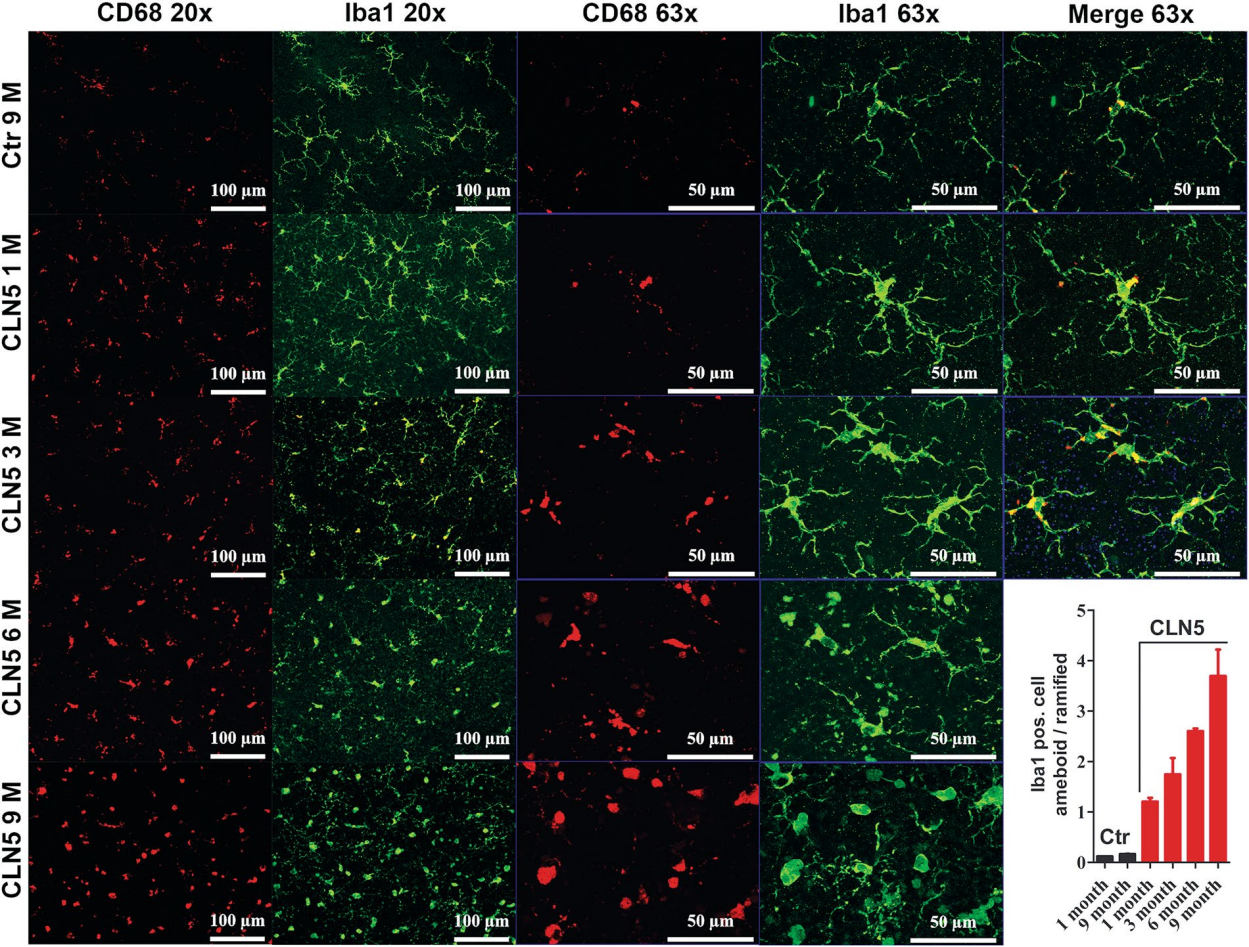
Early microglial infiltration and activation in CLN5 deficient mice retinas. Layout of representative z-stack images taken at the vicinity of ONL are shown for control and different aged CLN5 deficient retinal whole mount samples (rows, horizontal). CD68 (red, macrophage marker) and Iba1 positive (green, microglia marker) cells are shown with two different magnifications vertically in columns. Already at 1 month of age the CLN5 deficient retinas showed remarkably increased CD68 and Iba1 positivity and microglia cell morphology was changing from ramified form (inactive microglia) to a more amoeboid (active microglia) form. This pathological process progressed so that at 9 month of age very few Iba1 positive cells had ramified appearance. CD68 positivity was localized to the microglial cell body. Down-right corner: amoeboid – ramified cell morphology ratio was estimated by manual counting from Iba1 positive cells at 8 retinal locations and averaged for illustration.

We detected signs of microglia infiltration and activation in the CLN5 deficient retinal whole mount samples labeled with anti-Iba1 and anti-CD68 antibodies already at the age of 1 month (Fig. 6). CD68 is a marker for activated microglia, whereas Iba1 can be used to detect both resting and activated microglia^29^. Iba1 and CD68-positive cell numbers seemed to increase concomitantly with increased microglial cell body size. The appearance of the microglia began to be less branched and more amoeboid (see 63 x images in Fig. 6). Positive CD68 staining co-localized with the microglia cell body and the staining appeared more intense in cells having amoeboid morphology. The change of microglia cell morphology from ramified to amoeboid form seemed to be progressive with age in CLN5 deficient mice (Fig. 6, right-down corner). Few microglia with amoeboid appearance were detected in control samples (Fig. 6).

**Figure 7:**
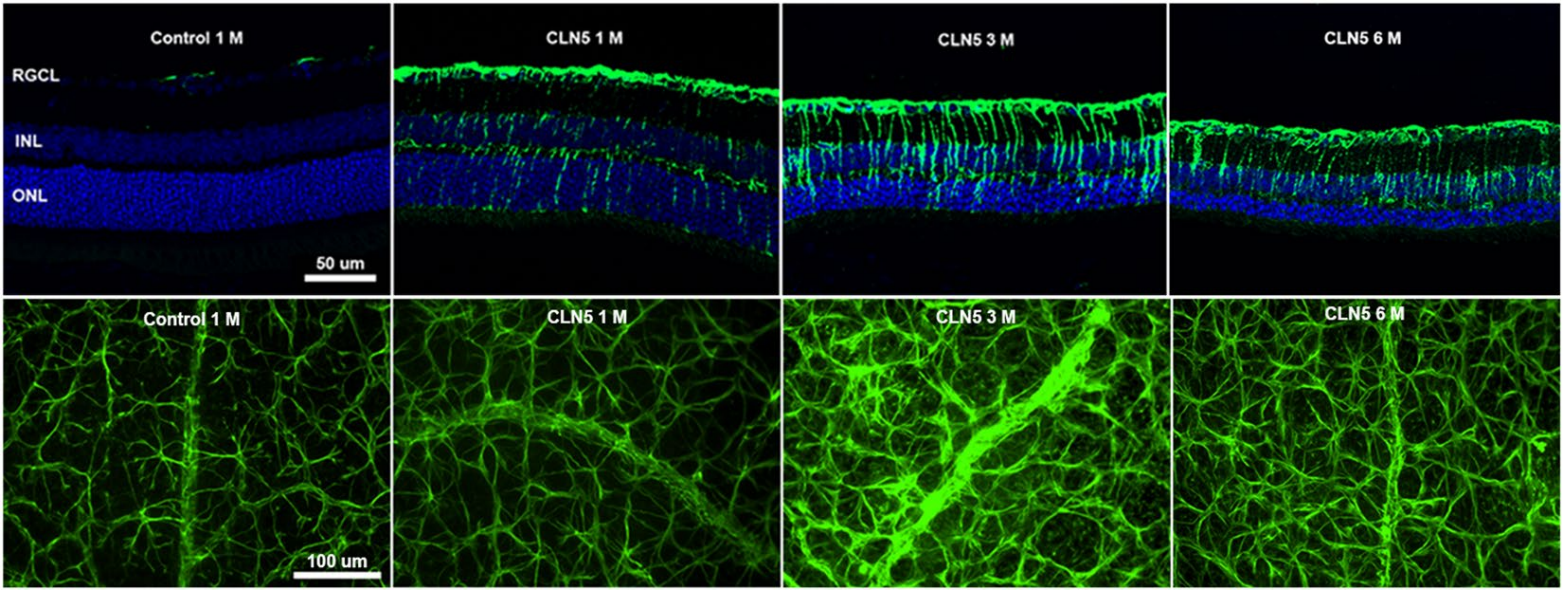
Müller cell gliosis and astrogliosis in CLN5 deficient mice retinas. Upper row: representative fluorescent z-stack images are shown from anti-GFAP (green, macroglia) staining with a DAPI counterstain (blue, cell nuclei). Astrocytes localize at the retinal nerve fiber layer, above the RGCL, horizontally and are expressing GFAP little in control samples. Müller cells are vertically oriented in the retina and do not express GFAP once inactivated. Already at 1 month of age, astrocytes and Müller cells were strongly activated in CLN5 deficient mice as shown by intense GFAP positivity. GFAP positive staining increased until 3 month of age. Lower row: anti-GFAP staining of retinal whole mounts. RGCL, retinal ganglion cell layer; INL, inner nuclear layer; ONL, outer nuclear layer; GFAP, glial fibrillary acidid protein.

Glial fibrillary acidic protein (GFAP) labeling in the retina reveals astrocytes and activated Müller glia cells. In CLN5 deficient retinal cross-sections, the GFAP staining was upregulated both in retinal astrocytes and Müller cells already at the age of 1 month (Fig. 7). The intensity of GFAP staining continued to increase until 3 months of age as shown both in retinal slice samples and whole mounts.

## Discussion

The first symptoms of CLN5 disease are usually motor clumsiness and visual impairment^28^, yet precise experimental data characterizing the retinopathy in CLN5 disease are lacking. However, ophthalmological examination has revealed signs of macular dystrophy and optic nerve atrophy^30^. In addition, the ERG response is known to become abnormal at an early age, and undetectable by the age of 7–9.5 years^31^. In CLN5 deficient mice, abnormal accumulation of autofluorescent storage material into the retinas has been reported, as well as visual impairment by a simple behavioral test^28^. In this study, we thoroughly characterized the retinopathy in CLN5 deficient mice.

In measurements of retinal function, we found that the scotopic c-wave of ERG was selectively diminished in amplitude in CLN5 deficient mice already at 1 month of age. The ERG c-wave is largely generated by a light-induced decline in subretinal potassium ion concentration and subsequent hyperpolarization of the apical membrane of the RPE^32^. It has been proposed that pathological changes in the RPE can be investigated by recording the c-wave^33–35^. However, the diagnostic usefulness of c-wave remains questionable as reflected by its relatively low utility. Moreover, recording of the c-wave is technically challenging and often leads to variable results, as was the case in the present study. Regardless of the problems related to the c-wave analysis, our data together with previous data from canine and ovine models of NCL^36–38^ indicate that the c-wave of ERG might be the most sensitive ERG component in detecting the first signs of NCL related retinal changes. It remains an open question, however, whether structural changes in the RPE causes these defects. In our analysis, the c-wave amplitude tended to decrease only diminutively with age in CLN5 deficient mice, and this trend towards decline could be attributed to progressive impairment in rod photoreceptor function^35^. Moreover, the appearance of RPE seemed normal at light microscope level in the early stages of the disease. Nevertheless, the c-wave could have some diagnostic utility in NCLs.

Functional changes arising directly from the neuronal retina occurred later in the disease progression than the observed change in the c-wave amplitude. We observed a trend towards decline in rod-dominant, scotopic a- and b-wave amplitudes at 2 months of age and the genotype difference became significant at 3 months of age. The ERG a- and wave reflect the activity of photoreceptors and bipolar cells, respectively^39, 40^. B-wave/a-wave amplitude ratio was higher in CLN5 deficient mice compared to control mice over the course of the study indicating that the pathology impaired the function of photoreceptors but not inner retinal cells^41^. In fact, the increase in b-wave/a-wave amplitude implies that the inner retina increased its sensitivity in response to a loss of input from photoreceptors. Similar sensitivity increase in the inner retina has been observed in mild-to-intermediate level photoreceptor degenerations induced by bright light exposure in mice and rats^41–43^. The increase in inner retina sensitivity might result from synaptic strengthening between the remaining photoreceptors and inner retinal cells^42^. However, it is well established that the inner retina undergoes negative remodeling in advanced photoreceptor degenerations, which may lead to depletion of RGCL and INL neurons among several other pathological changes^44, 45^. Indeed, we observed a statistically significant thinning of INL and IPL in CLN5 deficient mice occurring in an advanced stage of retinopathy. However, this change was minuscule compared to the fate of photoreceptors. It is notable here that although AF storage material accumulates in many parts of the retina^28^ only photoreceptors experienced pronounced destruction. Indeed, the causality of AF storage material localization and tissue damage have been virtually confirmed be inconsequential in NCLs^6^.

Deterioration of cone structure and function occurred several months later than rod function and thinning of the ONL. Notably, whereas 97% of the mouse photoreceptors are rods and the cones comprise only 3%^29^, thinning of ONL results from loss of rod nuclei rather than cone nuclei. In agreement with the photopic ERG response, the visual acuity derived from pattern VEP responses in photopic conditions was comparable between CLN5 deficient and control mice at 2 months of age, but seemed declined in CLN5 deficient mice at 6 months of age. This is somewhat consistent with behaviorally assessed visual decline in CLN5 deficient mice that started at around 4–5 months of age^28^. Moreover, von Schantz *et al*. showed marked neuronal loss in the primary visual cortex in CLN5 deficient mice at 4 month of age^46^. However, as the progression of decline in photopic visual acuity was modest in our study, and the latency of the VEP response remained normal, the vision loss in CLN5 deficient mice probably has a purely retinal origin. However, neuronal loss in the primary visual cortex of CLN5 deficient mice as shown by Von Schantz *et al*.^46^ may be in part explained by decreased input from retinal rod pathway that starts at a younger age.

The primary damage for rods over cones is common for *retinitis pigmentosa*, age-related macular degeneration (AMD), and light-induced toxicity^47, 48^. Rods are more sensitive to light-induced damage than cones in part due to greater accumulation of a toxic visual cycle-associated byproduct, all-*trans*-retinal, in rods^48^. In addition, the function of rods depends entirely on the output of 11-*cis*-retinal from the canonical visual cycle where photoreceptors and RPE interplay^49^. Cone photoreceptors also use 11-*cis*-retinal from the ‘rod cycle’ but may also utilize another RPE independent, yet poorly known, Müller cell-based ‘cone cycle’. Moreover, a recent study showed that living rods rather than cones are preferably phagocytized by activated microglia in retinal degeneration^50^. Collectively, our functional assessment suggested that the RPE-rod photoreceptor interface is the first retinal region affected in the CLN5 deficient mice retinas. This is followed by loss of rod photoreceptor nuclei, whereas cones, inner retinal neurons and photopic vision seem to be relatively long preserved. Correspondingly, preferential loss of signal from rods over cones has been detected in South Hampshire sheep, Tibetan Terrier dogs, PON dogs and CLN7 mouse model of NCLs^21, 51, 52^, well as in patients with juvenile type of NCLs^4, 53^.

The RPE must phagocytose 10% of the outer tips of POS every day for renewal, rendering retinal homeostasis extremely sensitive to insults in RPE’s phagocytic and autophagic capacity. POS renewal also maintains the visual cycle (regeneration of visual pigments) through a noncanonical form of autophagy^54^. A defect in any phase of the visual cycle can cause retinal degeneration^55^. Wavre-Shapton and coworkers recently showed defects in photoreceptor phagosome processing and autophagy in the RPE of a mouse model of CLN3 disease^14^. They found that the degradative compartments of autophagy and phagocytosis pathways could form normally in *Cln3* ^*deltaex1-6*^ mouse RPE, but the terminal phase of autophagic/phagocytic processing; lysosome fusion-mediated degradation and recycling of autophagosome and phagosome contents, was significantly impaired. Even more recently, Brandenstein *et al*. found increased expression of LC3-II and P62 positive aggregates, as well as increased expression of lysosomal cathepsins in microglia and neurons from CLN7 deficient mice brains, suggesting impaired lysosomal function and autophagy^15^. Furthermore, Thelen *et al*. showed increased levels of LC3-II and P62 in brain extracts of CLN6 deficient mice^18^. In line with the previous results, we detected increased levels of Beclin-1 and P62 proteins in the retinal extracts of adult CLN5 deficient mice by Western blotting. Beclin-1 is crucial for formation of autophagosomes and P62 marks poly-ubiquitinated proteins for selective autophagy^56, 57^. We also found a slight increase in LC3II/LC3I ratio in CLN5 deficient retinas, which may be attributable to increased autophagosome conversion from its cytosolic form to the membrane-bound form^58^. Instead, the level of lysosome-associated membrane-protein LAMP1, which is essential for phagosome-lysosome fusion^59^, was decreased. A probable explanation for the increased levels of Beclin-1 and P62 proteins, and LC-I to LC-II conversion, is a compensatory effect in response to a blockage in the last processing step of autophagy, the autophagosome-lysosome fusion. However, since our analysis was performed from 6-month-old neural retinas, the analysis is only a suggestive surrogate marker of what might be ongoing in the RPE in the early disease progress. The defect in RPE phagocytosis would be best investigated by electron microscopy from thin retinal sections, or alternatively from RPE cell culture by Western blotting.

Similarly to CLN6, the function of the CLN5 protein still remains unknown to date. However, it has been shown that CLN5 is a soluble protein localizing to the lysosomal compartment in wild-type samples *in vitro* ^60^, whereas CLN6 is a transmembrane endoplasmic reticulum (ER) protein^2^. Although CLN6 is retained in the ER and does not co-localize with lysosomal markers, lysosomal degradation is drastically affected in CLN6 deficient cell lines^61^. Correspondingly, mutations in the *CLN5* gene have been suggested to have an effect on lysosomal function^62^. Interestingly, the retinal phenotype of CLN5 deficient mice characterized here closely resembles that observed by others in CLN6 deficient mice. Bartsch *et al*. reported that ONL thickness was normal at eye opening but significantly declined already at 1 month of age in CLN6 deficient mice^9^. The loss of ONL nuclei was accompanied by strong microglial infiltration and activation, astrogliosis, and Müller cell gliosis from 1 month of age onwards in CLN6 deficient mice retinas^9, 12^. Instead, at two weeks of age, glial activation was not observed^9^. As the mouse eye opening occurs at 14–15 days postnatal^63^, the onset of visual pathology in CLN6 deficient mice may coincide with the activation of the visual cycle. We found clear signs of microglia infiltration and activation, Müller cell gliosis, astrogliosis and apoptotic photoreceptor nuclei in CLN5 deficient mice retinas already at the first time point studied, at the age of 1 month, although photoreceptor function was not yet diminished as assessed by ERG.

In conclusion, vision loss in CLN5 deficient mice seems to be primarily caused by pathological events in the RPE-rod photoreceptor interface, and subsequent death of rod photoreceptors. In addition to the CLN5 mouse model, findings from several other animal models of NCLs imply a similar pattern of pathological events in the retina^12, 21, 51, 52^. We speculate that the retinopathy in several forms of NCLs may be caused by failure of lysosomes to degrade phagosomes and autophagosomes. This speculation is based on previous reports by others proposing defective phagosome/autophagosome-lysosome fusion taking place in various tissues of NCL animal models, cell cultures or human patients^14–18^, and our tentative data indicating altered autophagy in the CLN5 deficient mouse retinas. The defect in phagocytosis/autophagy could be directly caused by a crucial NCL-associated protein dysfunction, or by protein-protein interactions. Of note, distinct CLN proteins may exert some of their actions through mutual interactions; for instance, CLN5 has been shown to interact with PPT1 (CLN1/CLN4), TPP1 (CLN2), CLN3, CLN6 and CLN8 proteins^64, 65^. From a therapeutic point of view, it seems that treatments that retain the normal functionality of CLN proteins by gene or stem cell therapies, or in some cases by enzyme replacement therapy (*e.g*. CLN2), may be the best strategy to treat NCLs both in the brain and the retina^7, 66^. However, since NCLs also affect peripheral organs negatively, systemic delivery of therapeutics that cross the blood-brain and blood-retina barrier would be optimal and worth exploring^67^. Finally, the study of the eye in NCLs is pivotal not only from an ophthalmological point of view – understanding the mechanisms of retinopathy in NCLs could help to disentangle the pathological basis of these devastating disorders in the rest of body.

## Material and Methods

### Animals

We utilized the CLN5 mouse model of late infantile Finnish variant of NCL generated originally in University of Helsinki^28^. The mouse model has been generated by disrupting exon 3 of the mouse *Cln5* gene, which results in a frame shift and premature stop codon in exon 4 of mouse *Cln5*. Both male (CLN5, n = 24; control, n = 26, in total) and female mice (CLN5, n = 22; control, n = 22, in total) were used. Age matched wild-type mice were used as controls. All the mice were maintained on the C57BL/6JRccHsd background. Animals were group-housed at 55% humidity, 22 °C temperature and with a 12 h / 12 h dark-light cycle (lights on 7 a.m to 7 p.m). Food and water were provided *ad libitum*. All experiments were conducted in accordance of Association for Research in Vision and Ophthalmology statement for the use of animals in research and the EU directive 2010/63/EU for animal well-being, using protocols approved and monitored by the Animal Experiment Board of Finland (ESAVI-2013-002477).

### Electroretinography (ERG)

The ERG methodology have been described in detail previously^68^. The animals were dark-adapted overnight for at least 12 h, all handling for ERG recordings were performed under dim red light, and mice were further dark-adapted in completely dark room for 7 min before stimulation. The light stimulation in scotopic conditions (rod-pathway) was done in ascending series in respect of stimulus intensity (−4.07, −3.23, −2.41, −0.90 and 0 log relative to max intensity), and the inter-stimulus interval (ISI) was increased accordingly (5 s, 10 s, 10 s, 20 s, 50 s, respectively). The maximum flash yielded ~4500 photoisomerizations (*R) per mouse rod (calibration described in ref. 69). After scotopic recordings a monitor in faraday cage was turned on to reflect grey light, at 17 lux, to the animal. After 10 min of light-adaptation photopic responses (cone-pathway) were recorded for a single light-intensity (log 0, 2 s ISI). Amplitude parameters for scotopic a-, b- and c-waves, and photopic b-wave were analyzed in this study. The baseline for ERG response was taken as the average amplitude between −100 to 0 ms before the stimulus onset. The a-wave amplitude was calculated from baseline to the trough of the first negative deflection after the stimulus onset. The b-wave was calculated from the a-wave trough to the peak of the first major positive wave. The c-wave amplitude was calculated between the maximum negativity between b- and c-waves (tail of PIII component in other words) to the maximum peak of the c-wave. Distinct cohorts of animals were tested for unrelated reasons at 1 months of age and 2–6 months of age.

### Visual evoked potentials (VEPs)

The VEP experiment was performed in awake, head-restrained animals following methods described earlier^70^. The experiment started with electrode assembly and head-stage implantation under isoflurane anesthesia when animals were 2 months of age. Stereotaxical coordinates for the screw electrodes above the primary visual cortex (V1) were −3.8 mm anterioposteriorly and −2.5 mm mediolaterally from bregma. Two screws were attached onto the frontal bone to serve as reference and ground electrodes. VEP stimuli consisted of vertical sinusoidal gratings displayed on a gamma-corrected monitor screen with reversing contrast at 1 Hz. Sweep VEP technique was used to determine the visual acuity of mice (technique reviewed in ref. 71). The VEP responses were recorded at 0.024, 0.048, 0.096, 0.192, 0.384, 0.48 and 0.576 cycles per degree (CPD) of visual angle. The pattern-VEP waveform comprised of a major positive wave occurring at around 55–80 ms from the stimulus onset followed by a negative deflection peaking at around 120–170 ms (see Fig. 1H). The VEP amplitude was calculated as the difference between the major positive (P1) and the major negative (N2) wave. If the negative wave was not pronounced, the VEP was calculated from the peak of the positive wave to the baseline. Visual acuity for each animal was determined by plotting a linear regression line through the amplitudes at different CPDs and extrapolating the voltage to 0 V. The VEPs were recorded at 2 and 6 months of age. To be precise, the first recording took place at 9 weeks of age due to recovery from surgery but is displayed as 2-month for presentation clarity and consistency.

### Tissue collection

Retinal samples were collected from mice perfused with heparinized saline. The mice were deeply anesthetized by an overdose injection of Avertin (tribromoethanol 625 mg/ml in 40% tertamylalcohol; 0.02 ml/g, i.p.) and perfused through the left ventricle for 3 min at 20 ml/min prior to the eyes being enucleated. For immunohistochemistry, one eye from each mouse was fixed in 4% paraformaldehyde (PFA) overnight, washed in phosphate buffered saline (PBS, 0.1 M, pH 7.4) overnight, and finally embedded into paraffin blocks. The second eye was fixed in 4% PFA for 3 h. The cornea was cut away following the orientation of *ora serrata* and the lense was removed from the eye cup. The retina was dissected from the eyecup and prepared as whole mount sample. For Western blot analyses, the eyes were quickly enucleated after perfusion and placed on a petri dish into ice-cold PBS droplets. The retina was dissected out as described above. The fresh retinal samples were placed in Eppendorf tubes, snap-frozen with liquid nitrogen and stored at −70 °C until further analysis.

### Western blot

Protein concentration in retinal tissue homogenates collected from 6-month old mice was determined with Pierce BCA Protein assay kit (Thermo Cat, No. 23225). Equal amounts of protein were loaded on 10% SDS-PAGE gels and separated using a Mini-Protean 3 (Bio-Rad) device at 200 constant voltage. For immunoblotting, proteins were transferred onto Hybond P membrane (GE Healthcare) in a Mini TransBlot (Bio-Rad) chamber. Membranes were blocked in 5% skimmed milk solution in PBS (containing Tween) and incubated with primary antibodies against P62, Beclin-1, LC3b, LAMP1 (see Table 1 for spesifications) and β-actin (mouse monoclonal, 1:5000 dilution, Sigma, St. Louis, MO). The blots were developed by incubating with anti-rabbit HRP-labelled secondary antibodies (GE Healthcare, 1:2000) and Pierce ECL Plus WB substrate (Thermo Cat.No. 32132), or anti-mouse Cy5-labelled antibodies (Jackson ImmunoResearch, 1:1000). The membranes were visualized on Storm 860 Fluoroimager (GE Healthcare) and quantified with ImageQuant software (GE Healthcare).

**Table 1:** Details of antibodies used in immunohistochemistry (IHC) and Western blot (WB).

Antibody	Host	Source	Dilution	Spesificity
Anti-GFAP	Rabbit	DakoCytomation	1:10 000 (IHC)	Macroglial marker
Anti-Iba1	Rabbit	Wako	1:500 (IHC)	Microglial marker
Anti-CD68	Rat	AbD Serotec	1:1000 (IHC)	Macrophage marker
Anti-Beclin1	Rabbit	Thermo Scientific	1:1000 (WB)	Autophagy marker
Anti-P62	Rabbit	Cell Signaling	1:1000 (WB)	Autophagy marker
Anti-LC3b	Rabbit	Cell Signaling	1:1000 (WB)	Autophagy marker
Anti-Lamp1	Rabbit	Abcam	1:1000 (WB)	Lysosomal marker
Anti-M/L opsin	Rabbit	EMD Millipore	1:1000 (IHC)	M/L opsin marker

### TUNEL assay

Terminal deoxynucleotidyl transferase dUTP nick end labeling (TUNEL) staining was performed as per manufacturer’s instructions (Sigma Aldrich, Cat no. 11684795910). Briefly, the paraffin embedded sections were deparaffinized in xylene and descending grades of alcohol. Next, antigen retrieval was performed in a microwave oven at 750 W for 1 min followed by rapid cooling in PBS. The test slides were finally treated with freshly prepared TUNEL reaction mixture followed by incubation in a humidified chamber at 37 °C for 1 h. The positive control was treated with DNAase I (Sigma Aldrich, Cat no. 9003–98–9) before application of TUNEL reaction mixture and the enzyme solution was omitted from the negative control. An open-source imaging platform (https://imagej.nih.gov/ij) in ImageJ program was used to count DAPI positive cells in the ONL. Briefly, images taken with a 20 x magnification were converted to gray scale, straightened and analyzed within 6 counting windows measuring approximately 370 µm in width. TUNEL positive cells were counted manually online across the entire ONL using a 20 x magnification.

### Histology, morphometry, immunohistochemistry and microscopy

The paraffin-embedded eyes were cut into 5 µm sections with a microtome. For thickness analysis of retinal layers, retinal panorama pictures were constructed from hematoxylin & eosin stained samples. The length of the retina was manually determined with a ruler tool in Zen 2012 software (Zeiss, Jena, Germany). Marks were digitally drawn to the panorama picture into locations of 25%, 50% and 75% distance from optic nerve head (ONH) in relation to the end tip of the retina at *ora serrata* to both sides of the retina. The thickness of the ONL, INL and IPL were measured at the vicinity of the six predetermined locations using the ruler tool. The measurements at 25%, 50% and 75% locations from both sides of the retina were finally averaged and the average was used for statistical analysis. The samples were inspected by a fluorescent light-microscope (Zeiss Imager M2, Zeiss, Oberkochen, Germany) and images acquired with a digital camera for light-microscope (AxioCam ERc5s, Zeiss, Oberkochen, Germany) or fluorescent microscope (AxioCam MRm, Zeiss, Oberkochen, Germany).

For immunohistochemistry (IHC), the sections were deparaffinized, rehydrated and washed in PBS (0.1 M, pH 7.4) or tris-buffered saline (TBS, pH 7.6). Antigen retrieval in GFAP staining was performed by boiling sections in 0.05 M tri-sodium citrate dihydrate (pH 6.0) at 90 °C for 5 min followed by washing 3 × 5 min in PBS/TBS. The sections were blocked in 3% bovine serum albumin (BSA) or 10% normal goat serum (NGS) for 1 h and then incubated in primary antibody (diluted to 3% BSA or 5% NGS in buffer solution) with mild shaking overnight. Then the sections were washed for 3 × 5 min in a buffer solution and incubated with a fluorescent secondary antibody for 3 h (Anti-Rat AlexaFluor 568 nm, dilution 1:500; Anti-rabbit AlexaFluor 488 nm, dilution 1:500, Life Technologies). Finally, the sections were washed again for 3 × 5 min, air dried for 5–20 min and mounted using Vectashield mounting medium (Vector Laboratories, Burlingame, CA, USA) with fluorescent DAPI (nuclear stain). The whole mount retinal samples were stained otherwise similarly but the samples were freely floating and 0.25% Triton X-100 was added to washing and incubation solutions to increase tissue permeability. The primary antibodies used for IHC and Western blot are displayed in Table 1.

Three sample glasses of each genotype and age group were stained and imaged using a fluorescent microscope. AF imaging of retinal sections (Fig. 3) was performed from a single depth plane using a 20 x objective and green fluorescent filter. Other fluorescent images from retinal slices (Figs 5 and 7) were obtained using Z-stack and apotome attached to the microscope. Multiple images for z-stack were acquired with an imaging interval of 0.5 µm for a total depth of 5 µm. The exposure was set automatically by the imaging software (Zen 2012). Whole mounted retinas for microglia inspection were imaged similarly using the Z-stack, except that the imaging depth was 10 µm. The images of Iba1/CD68 double-labeled whole mounts were taken at the depth of OPL. Iba1-positive cells demonstrating either ramified or amoeboid morphology were manually counted online at 8 retinal locations (4 locations at central and 4 at peripheral retina) with a 40 x water immersion objective. The ONH was used for orientation and the counting window was moved superiorly, inferiorly, temporally and nasally. The counting window was 225 µm in width and 175 µm in height. The microglial cells were counted through the depth of the whole retina. Finally, the ratio between amoeboid and ramified microglia was calculated. Whole mount retina images in Fig. 7 and Supplementary Figs 1 and 2 were taken without a z-stack. M/L cone outer segments (COS) were counted manually using a 63 x objective, stereology microscope (Axioplan, Zeiss, Oberkochen, Germany) and StereoInvestigator 7 software (MBF Bioscience, Williston, VT, USA). The size of the counting window was 75 µm × 75 µm and COS were counted from 19 locations on average. Median density of COS (the counting windows that had 25% of lowest and highest counts were omitted from analysis) was used in statistical comparison.

### Statistical analyses

All statistical analyses were performed using GraphPad Prism version 7 (La Jolla, CA, USA). The group means in the ERG were compared using two-way analysis of variance (ANOVA) followed by Bonferroni’s multiple comparisons tests. The VEP data were analyzed otherwise similarly except that ANOVA for repeated measures was used. The retinal layer thickness was assessed by one-way ANOVA followed by Tukey’s post-hoc test. The western blot data were analyzed by Student’s t-test assuming equal variances (tested by Levene’s test). Group means ± SEM are displayed. Threshold for statistical significance was set at p < 0.05.

## Electronic supplementary material

Supplementary Information
