# Supplementary material for: Retinal Degeneration In A Mouse Model Of CLN5 Disease Is Associated With Compromised Autophagy
Source: Sci Rep. 2017 May 9;7:1597. doi: 10.1038/s41598-017-01716-1 (PMC5431647; doi:10.1038/s41598-017-01716-1)
Supplement: Supplementary file 1 — Supplementary Information [file 41598_2017_1716_MOESM1_ESM.doc]

**SUPPLEMENTARY MATERIAL FOR:**

**RETINAL DEGENERATION IN A MOUSE MODEL OF CLN5 DISEASE IS ASSOCIATED WITH COMPROMISED AUTOPHAGY**

Leinonen Henri^1#*^, Keksa-Goldsteine Velta^1^, Ragauskas Symantas^2^, Kohlmann Philip^1^, Singh Yajuvinder^1^, Savchenko Ekaterina^1^, Puranen Jooseppi^2^, Malm Tarja^1^, Kalesnykas Giedrius^2,3^, Koistinaho Jari^1^, Tanila Heikki^1^, Kanninen Katja M^1*^.

^1^A.I. Virtanen Institute for Molecular Sciences, University of Eastern Finland, Kuopio, Finland.

^2^Experimentica Ltd., Kuopio, Finland.

^3^Research and Development Centre for Ophthalmic Innovations (SILK), Department of Ophthalmology, University of Tampere, Tampere, Finland.

**
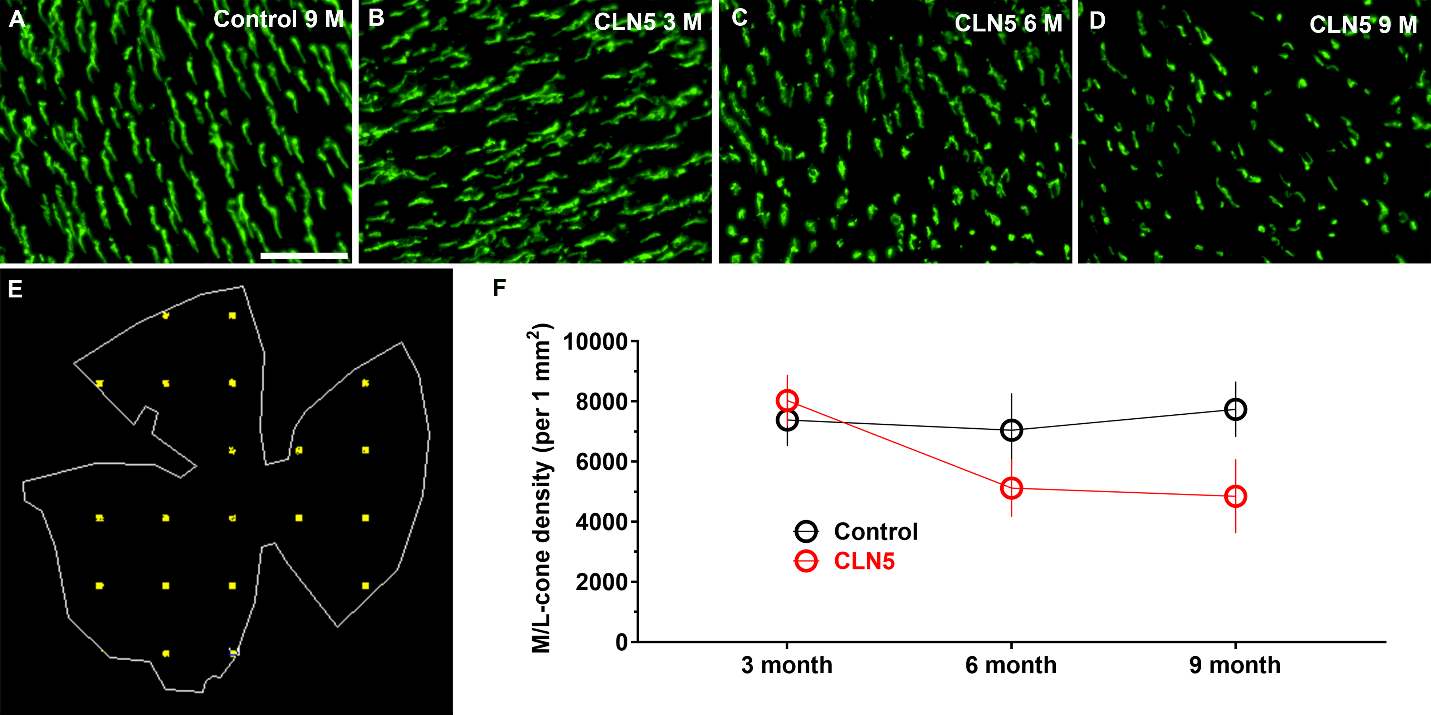
**

**Supplementary Fig. 1.** Immunohistochemical staining of retinal whole mount samples show shrinking of medium-to-long wavelength (M/L) cone photoreceptor outer segments (COS) and a tendency to decreased cone count at an advanced disease state. COS were stained by anti-M/L opsin antibody. A-D: representative retinal images were taken with a 40 x magnification. E: a schematic image illustrating an example of COS counting locations from retinal whole mounts. F: median cone density was comparable in control and CLN5 retinas at the age of 3 months (n=3 per genotype). As the disease progressed the M/L cone density tended to decline in CLN5 deficient mice as compared to controls (genotype effect in ANOVA: F[1,15]=4.1, p=0.06; 3-6-month: n=3-4 per genotype; 9-month: n=4 per genotype).

**
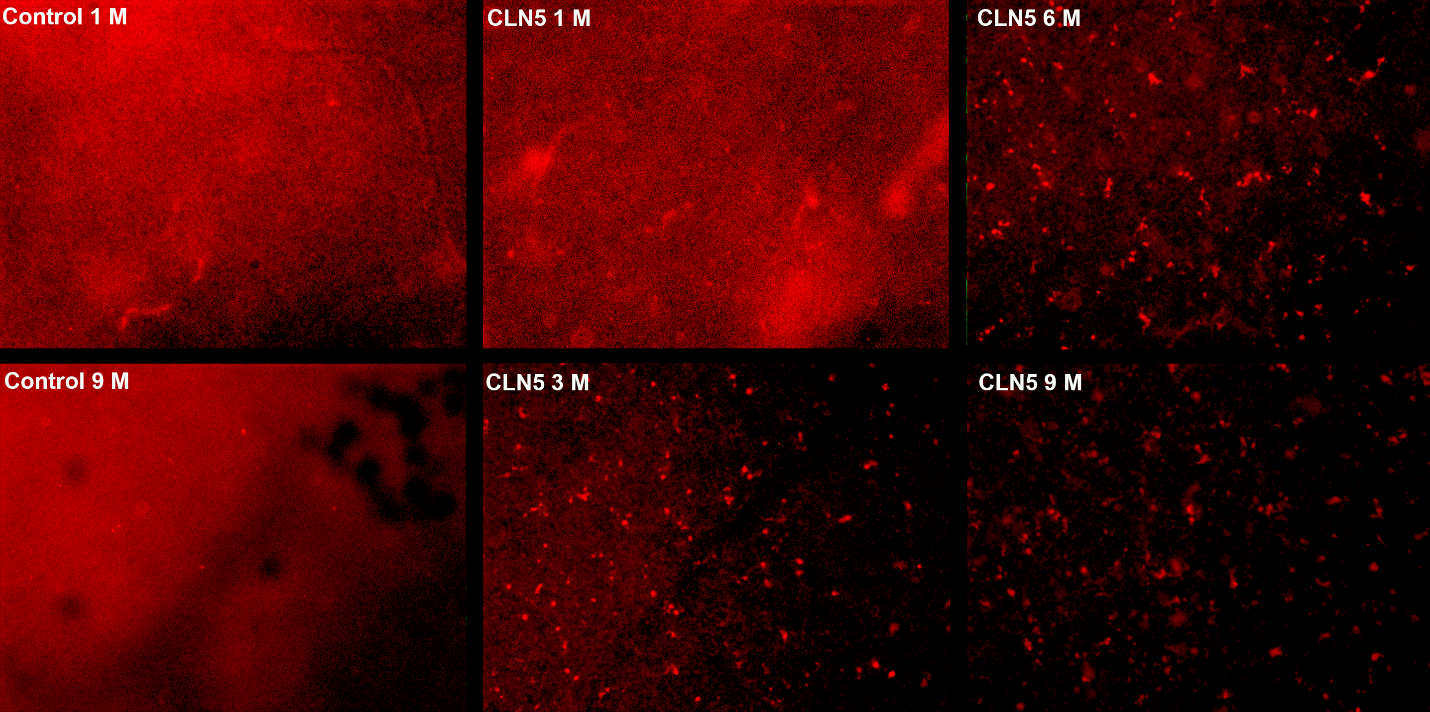
**

**Supplementary Fig. 2.** Accumulation of autofluorescent (AF) aggregates in CLN5 deficient retinas. Whole mount retinas were inspected with a fluorescent microscope and AF was revealed using a red filter. Distinct AF aggregates were apparent in CLN5 deficient samples at 3, 6 and 9 months of age, but not yet at 1 month of age. Note that RPE is removed from whole mount retinal sample. The images were taken from the same x-y axial location as whole mount images shown in Fig. 7. AF aggregates were found most abundantly approximately 30 µm below the nerve fiber layer (*i.e.* where astrocytes reside) and approximately 12 µm above photoreceptors (as revealed by DAPI staining, data not shown) corresponding to outer plexiform layer.


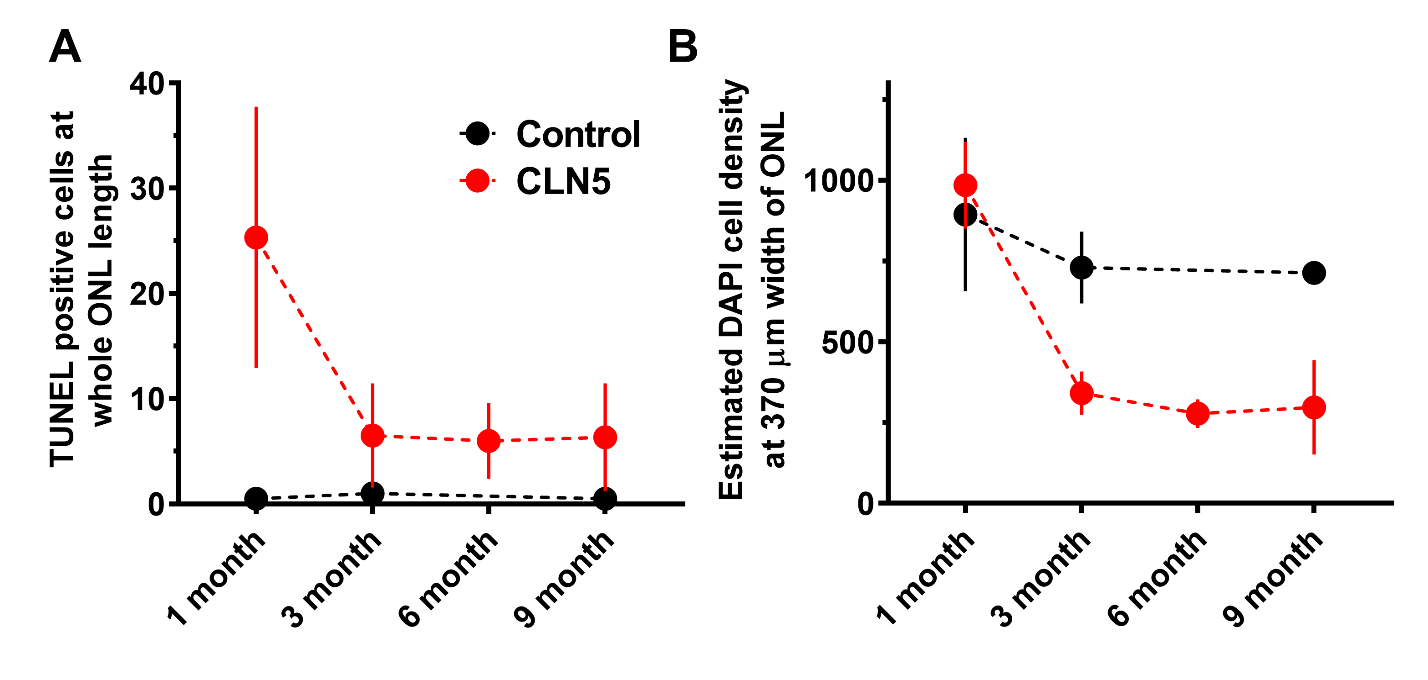


**Supplementary Fig. 3.** The count of TUNEL positive cells in the ONL seemed to decline together with decreasing ONL nuclei density in CLN5 deficient mice. A: TUNEL positive cell count at the outer nuclear layer (ONL, photoreceptors) as measured manually from the whole retinal length. B: Computerized estimate of cell nuclei (DAPI) density at 370 µm width of ONL (average of 6 counting windows). N=3 per genotype per age-point (note that age-point 6 month is missing from controls). Statistical analysis was not performed due to small sample size.
